# Untypical symptoms for rather uncommon surgical entities: report of two rare cases of secondary intussusception in children (a case report)

**DOI:** 10.11604/pamj.2020.37.277.26829

**Published:** 2020-11-26

**Authors:** Godosis Dimitrios, Mouravas Vasileios, Lambropoulos Vassilis, Kepertis Chrysostomos, Anastasiadis Kleanthis, Spyridakis Ioannis

**Affiliations:** 1 2^nd^ Paediatric Surgery Department, Papageorgiou General Hospital, Aristotle University of Thessaloniki, Thessaloniki, Greece

**Keywords:** Case report, secondary intussusception, non-Hodgkin lymphoma, inflammatory myofibroblastic tumor

## Abstract

Intussusception in infants and children represents a relatively usual challenge for the pediatric surgeon. However, the incidence of lymphoma of the small intestine or inflammatory myofibroblastic tumor, acting as a lead point for invagination, are rather rare. We hereby present two cases of secondary intussusception, with the aforementioned lead points.

## Introduction

Pediatric lymphoma represents almost 15% of all pediatric malignancies [1]. Furthermore, Inflammatory Myofibroblastic Tumors (IMTs) represent a very rare condition, categorised as intermediate neoplasms, according to World Health Organization (WHO). Intussusception occurs at about 34-90/100 live births, with considerable regional variability, according to recent reports [2]. Nevertheless, the combination of a small intestinal lymphoma acting as a leading point for secondary intussusception is a rather rare condition. The latter is presented as a case of a young boy admitted to our emergency department for abdominal pain.

## Patient and observation

### Patient 1

**History:** an eight-year-old boy with prior uncomplicated medical history was admitted to our pediatric surgery emergency department complaining about abdominal pain. In particular, symptoms began 2 days prior to his admission to our faculty, having intermittent characteristics and located particularly to the mesogastrium. The patient had no other accompanying symptoms, such as fever, nausea or vomiting. He had a normal defecation during the day of his admission.

**Physical examination:** physical examination revealed tenderness in the periumbilical area and right iliac fossa, with no palpable mass or distension. He also presented with vital signs within normal limits, according to his age. Laboratory exams at the emergency department resulted in leukocytosis with neutrophilic shift (16.6X10^3^/u, 86% neutrophils, 7.6% lymphocytes, 5.9% monocytes and 0.2% eosinophils. He also had elevated C-reactive protein levels (6.61mg/dL, normal levels <0.8mg/dL), with normal procalcitonin levels. An abdominal ultrasound examination and erect abdominal X-ray were not indicative for certain pathology. Due to his clinical condition, an admission to our department was proposed to his parents, for surgical investigation.

**Figure 1:**
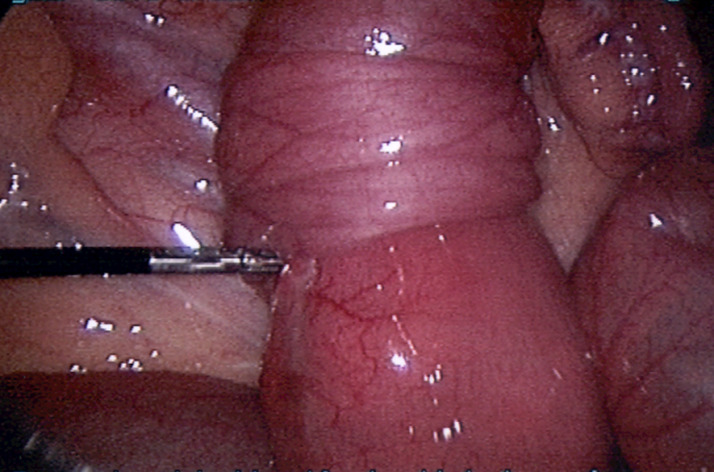
laparoscopic approach revealed an ileoileal intussusception, approximately 40cm from the ileoceacal valve

**Figure 2:**
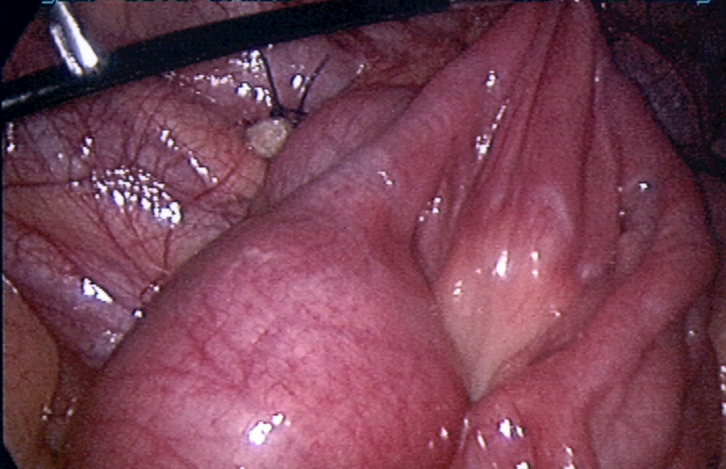
a laparoscopic disintussusception approach was performed, with no signs of ischemia or perforation after the reduction of the small bowel

**Management:** the patient was guided to the operating theatre and an exploratory laparoscopy was initiated. Inspection of the peritoneal cavity revealed a macroscopically inflammatory appendix, with serosal hyperaimia. A laparoscopic appendicectomy was performed. Furthermore, when the small intestine was inspected, an ileoileal intussusception was also noted, approximately 40cm from the ileoceacal valve (Figure 1). A laparoscopic disintussusception approach was performed. Reduction of the small bowel revealed a palpable intraluminal mass that acted as a lead point for invagination, with no signs of ischemia or perforation (Figure 2). After a confined elongation of the umbilical incision, the problematic ileum containing the mass was exerted through the umbilicus. An in situ resection with an end-to-end ileoileal anastomosis was carried out, obtaining a 15cm histologic specimen for investigation.

**Outcome:** histopathology examination resulted in an acute empyematous appendicitis with hyperplasia of lymphoid tissue and also lymphatic cells inflitration. In terms of the resected ileal segment, the intestinal wall appeared microscopically infiltrated with atypic large lymphoid cells with big nuclei and distinct nucleoids, in a diffuse manner. Lymphoid aggregations of the type of “starry sky” with lots of macrophages were diffusely noted. The Ki67 proliferative index was 85%. Tumor cells were also positive for CD20, CD79a and CD10. They were negative for CD23, CD30 and bcl-2. Surgical incision limits were not infiltrated by tumor cells. The child had an uneventful postoperative period, during his stay in hospital. He was discharged from our department on the 6^th^ postoperative day, having been referred to pediatric oncologist for further management.

### Patient 2

**History:** a 13-year-old girl was admitted from a regional hospital to our department with a prior radiologic diagnosis of intussusception at the small intestine.

**Physical examination:** the patient was complaining about diffuse abdominal pain. She was afebrile, with normal vital signs and normal laboratory exams during her investigation at the emergency department. A new ultrasound just confirmed the diagnosis.

**Management:** the patient underwent laparotomy where, a jejunojejunal intussusception was found 60cm distally to the ligament of Treitz. At reduction, a tumor was found as a lead point. A 15cm long segment of jejunum was resected and end-to-end anastomosis was performed. The wall of the excised bowel contained two well circumscribed, solid, partly grayish-white and partly brownish-red lesions measuring 6.4cm and 1.2cm respectively.

**Outcome:** histologically both lesions were similar, containing fibroblastic or myofibroblastic spindle cell proliferations with an oedematous background of collagen fibers and vessels. The lesions also contained varying degrees of inflammatory cell infiltrations, predominantly plasma cells, lymphocytes and macrophages with occasional eosinophils. Mitoses were rare. The neoplasms extended all through the jejunum wall, from the mucosa to the mesenterium. Immunohistochemically the fibroblastic or myofibroblastic spindle cells were positive for vimentin, weakly positive for CD-34 and negative for CD-117, CD-68, LCA, S-100, AE1/AE3, DOG-1 and SMA. Ki-67 proliferative index was 2-3%. These findings were consistent with a small bowel Inflammatory Myofibroblastic Tumor (IMT). The young patient recovered easily after the surgery. Up to date, 2.5 years postoperatively, follow-up laboratory studies and Computed Tomography (CT) scan are normal.

## Discussion

Intussusception represents one of the most frequent causes of acute bowel obstruction in infants and toddlers and the second most common cause of acute abdominal pain in infants and preschool children after constipation. As Gross stated in 1953: “there are few illnesses in which the clinical history and physical findings are more suggestive of the correct diagnosis” [3]. Demographically, it occurs with an incidence of approximately 1 to 4 in 2000 infants and children throughout the world. A 2: 1 to 3: 2 ratio (male dominance), with a 75% of the cases presented within the first 2 years of life and a 90% of cases in children within 3 years of age. More than 40% are noted between 3 and 9 months of age [3].

The pathologic anatomy of each intussusception follows a certain path: the proximal invaginated bowel (intussusceptum) carries its mesentery into the distal recipient bowel (intussuscipiens). Then, mesenteric vessels are angulated and compressed between the layers of the intussusceptum, causing local oedema, venous compression, congestion and stasis, leading to the outpouring of mucus and blood from the engorged intussusceptum, the classic red currant jelly stool. If left unabated, intussusception leads to bowel necrosis, in most cases about 72 hours after the beginning of the invagination [3].

Regarding the cause of intussusception, up to 95% of the cases are categorised in the so-called “idiopathic” type, which in fact represents an invagination of a thickened bowel wall lymphoid tissue (Peyer patches) acting as an actual (non-pathologic) lead point. The incidence of intussusception from a pathologic lead point in infants and children ranges from 1.5% to 12% [4]. Most common causes are an inverted Meckel´s diverticulum, intestinal polyps and duplications. Other less common causes are periappendicitis, appendiceal stump, inversion appendectomy, appendiceal mucocele, local suture line, massive local lymphoid hyperplasia, ectopic pancreas, abdominal trauma, benign tumors (adenoma, leiomyoma, carcinoid, neurofibroma, hemangioma) and malignant tumors (lymphoma, sarcoma, leukemia). It must be also noted that most of the intussusceptions arising from a pathologic lead point manifest as ileoileocolic, with a smaller percentage being jejunojejunal, ileoileal, ileocolic, appendicocolic, cecocolic and colocolic. With regard to a malignant tumor acting as a pathologic lead point, lymphomas in general present as intussusceptions at a frequency of 17.5% and often resolve with contrast enema [3].

However, secondary intussusception with a pathologic lead point of non-Hodgkin lymphoma (NHL) tumor is a rather rare condition [5], although there has been a recent publication with two such pathological sites at the same time, one as intussusception and the other as mandibular oedema [6]. Noteworthy is also the fact that Burkitt´s lymphoma has been proved responsible for cases of chronic intussusception among children [7,8]. However, only 10% of NHL is confined to the gastrointestinal tract [9]. Factors predisposing for this type of intestinal lymphoma in children include immunodeficiency conditions, inflammatory bowel disease or malabsorption syndromes [10,11]. High suspicion is required from surgeons, as an undiagnosed NHL lymphoma has non-specific clinical presentation and can easily mimic acute appendicitis [12,13]. In our case, clinical manifestation was more suggestive for a complicated appendicitis (in terms of diffuse abdominal pain) and was neither favoring any systematic signs of NHL nor typical clinical and radiological signs or symptoms of intussusception.

Regarding IMT, it is a neoplasm of myofibroblastic spindle cells with inflammatory infiltration. It mostly occurs between 2-16 years of age. Such lesions have been reported in a variety of organs including the colon, lung, bladder, spleen, breast, pancreas, liver, spermatic cord, prostate, peripheral nerves, soft tissue and orbit. Small bowel localizations are particularly rare [14] and usually solitary. They recur locally, manifest systemic symptoms, but rarely present malignant transformation and are classified as intermediate neoplasms in the World Health Organization histological typing [15]. The cause of IMF tumors is unclear; many authors have postulated a post-inflammatory process. Differential diagnosis of IMT includes inflammatory fibroid polyp, fibromatiteroid and cyclosporin-A have been used as treatment modalities, but surgical resection is considered as treatment of choice [16]. In terms of recurrence, published bibliography proves that most cases have been reported as local remanifestation in mesentery and retroperitoneum [17]. However, complete surgical removal ensures almost zero recurrence rates, especially when multiple lesions are not observed. Keeping in mind that most recurrences, when presented, are manifested 1 year after initial surgery [18] and that the most common nature of such lesions is rather benign, a follow-up plan of CT scans every 6 months for the following two postoperative years is recommended.

## Conclusion

A clinical pediatric surgeon has to raise high suspicion in terms of some rather rare causes of secondary intussusception, especially regarding cases of benign or malignant tumors acting as a lead point, as such cases require different postoperative follow-up than usual.

**Ethical considerations:** patients´ parents provided written informed consent for their children´s surgical operation and agreed with also written consent for the use of history, laboratory-radiological exams and images without identifying information.
